# Exploring the effect of the Group Size and Feedback of non-player character spectators in virtual reality exergames

**DOI:** 10.3389/fpsyg.2023.1079132

**Published:** 2023-05-18

**Authors:** Wenge Xu, Kangyou Yu, Xuanru Meng, Diego Monteiro, Dominic Kao, Hai-Ning Liang

**Affiliations:** ^1^DMT Lab, Birmingham City University, Birmingham, United Kingdom; ^2^Department of Computing, Xi'an Jiaotong-Liverpool University, Suzhou, China; ^3^Digital Engineering School, ESIEA, Laval, France; ^4^Department of Computer and Information Technology, Purdue University, West Lafayette, IN, United States

**Keywords:** virtual reality, exergames, virtual spectator, spectator feedback, non-player characters

## Abstract

Despite the widespread interest in leveraging non-player characters (NPCs) to enhance gameplay experiences, there is a gap in understanding of how NPC spectators (i.e., those virtual characters in the scene that watch users' actions) affect players. For instance, the impact of NPC spectators' presence and feedback on players' performance and experience has not been studied, especially in virtual reality (VR) exergames. This paper aims to fill this gap and reports two user studies that assess their effect on such games. Study 1 explored the impact of having NPC spectators present and their feedback available in a gesture-based VR exergame and found having NPC spectators and their feedback could improve players' game performance, experience, and exertion. Based on Study 1's results, we further explored two characteristics of the spectators—their group size (small/large) and their feedback (with/without). The results show that (1) a large spectator number is more helpful since it improves the overall game experience (higher competence, flow, immersion), increases AvgHR% (the average heart rate percentage divided by the maximum heart rate), and enhances performance (improved players' combo performance and increased gesture success rate for particular gesture); (2) spectator feedback is instrumental in improving players' performance (higher gesture success rates, more combos performed successfully, more monster's combos prevented), enhancing game experience (positive affect, competence, flow, and immersion), and reducing negative game experience, increasing exertion (AvgHR% and burned more calories). Based on the results, we derived two main design recommendations for VR exergames that could pave the way for improving gameplay performance and game experience, especially among young adults.

## 1. Introduction

Non-player character (NPC) spectators have long been used in sports videogames (e.g., NBA 2K^1^, FIFA^2^, Creed: rise to glory^3^) and have played an essential role in these commercial games. With the arrival of virtual reality (VR), which relies on body motions, there has been an emphasis on developing games that use physical movements as a way to make exercising fun and are tailored for different age groups (Xu et al., 2021a,b, 2022). Recently, Haller et al. (2019) have employed spectators NPCs in a VR cycling game to explore whether having NPC spectators next to the cycling route to cheer and clap for the player could improve the player's performance and maintain intrinsic motivation. Their results showed that the virtual crowd feedback increased players' performance (cycling speed) and heart rate during the game. However, whether these NPC spectators can improve gesture-based exergame experiences is still unknown. Therefore, this work first explores whether NPC spectators (their presence and feedback) could impact players' performance and experience in a gesture-based VR exergame.

While Haller et al. (2019)'s work shows positive results, it is preliminary and limited (e.g., they used only a stationary bike application). This research further investigates other characteristics of NPC spectators in gesture-based VR exergames, which tend to be more common to have a spectator audience. One factor is the group size (or number) of NPC spectators in the environment, which has been widely studied in different fields but not in VR exergames. It has been found that human spectators have a negative impact on players' performance in real-life sports settings when the number of spectators is large, for instance, at the beginning of the matches where the situation is non-critical (Böheim et al., 2019). The effects of NPC spectator group size have also been studied within VR environments for public speaking tasks, where the literature suggests that a smaller number of spectators leads to significantly higher stress responses (in particular in users' heart rates) than a larger number of spectators (Mostajeran et al., 2020).

In addition, feedback provided by human spectators has also played an essential role in games. The *social cognitive theory* suggests that encouraging feedback is helpful to inform recipients that their performance has achieved a certain standard and they made good progress (Ilgen et al., 1979; Latham and Locke, 1991), which in turn could result in more positive performance outcomes and improved self-efficacy and confidence (Bandura, 2001). This theory has also been studied in exergames. A previous study (Kim and Timmerman, 2016) suggests that players who receive highly supportive feedback (e.g., with messages like “You are doing great! Keep it up!” and “Wow! You are making good progress!”) had a greater exergame experience (e.g., they enjoyed the game and activity more) than those who receive low supportive feedback (e.g., “You can do better than this” and “Well, I do not think you are making very good progress”). One important aspect of this research is that the feedback was provided by an actual human researcher and not via spectator NPCs, which can be more practical and give more flexibility to game designers (e.g., to consider different spectator types and sizes).

In this work, we first investigate the effect of NPC spectators by comparing players' performance and experience of a VR gesture-based exergame in a condition where there is no NPC spectator to a condition where there are a small group of spectators that can provide support to the player and the opponent. Results suggest that participants had better performance (performed more combo moves and better success rates in some gestures) and experience (positive affect, competent, flow, and immersion) when the NPC spectators were present and their feedback was provided. Then we further explore the effect of the NPC spectator group size (small and large) and their feedback (with and without) in the second study. Based on the results, we provide two design recommendations that can support framing VR exergames to include NPC spectators and their feedback.

## 2. Related work

### 2.1. Effect of NPC spectators in digital games

NPC Spectators have long been used in videogames, and some studies have explored their effect. Emmerich and Masuch (2016) conducted a study to investigate the presence of a virtual agent (present and absent) and display type (computer monitor and VR HMD) and showed that the gameplay performance was worse in VR HMDs when a virtual agent was present but not in the computer monitor. Later, they attempted to understand the impact of a real observer and virtual agents on games under three conditions: (a) the presence of the experimenter, (b) the presence of a virtual agent (i.e., controlled by a computer program), and (c) the control condition where the participant was alone. They found gameplay performance was not influenced by the presence of any social entity (Emmerich and Masuch, 2018). A recent study suggests that purposefully integrating observation and surveillance based on text phrases shown in games can improve players' performance, experience, and motivation (Kao, 2021). NPC Spectators have also been employed in VR cycling games, where Haller et al. (2019) have found that having NPC spectators cheer and clap for the player could improve player performance and maintain high intrinsic motivation. Although incorporating the effect of NPC spectators has been gaining attention, it is still seldom studied, and its impact has not been evaluated in other types of exergames (e.g., gesture-based). A deeper understanding of their characteristics (group size, feedback) and their effect is also necessary to understand how to integrate them into VR exergames.

### 2.2. Effect of spectator group size

Being in the spotlight can be frightening to many, especially in front of a large number of spectators. Prior research from sports sciences suggests that an audience could negatively impact the home team's performance at the beginning of games, which is known as the “home choke” effect (Baumeister, 1984; Goldman and Rao, 2012; Böheim et al., 2019). Moreover, studies on the effect of spectator group size have shown that nervousness increases proportionally according to its size and status (Latané and Harkins, 1976). However, the “home choke” effect may not be applicable to other contexts and general users who exercise for fun. For instance, research on public speaking tasks in VR suggests the opposite effect. Mostajeran et al. (2020) have found that participants had a higher stress response (in particular heart rate) when performing a public speaking task in front of a small number of spectators than a large one. In this study, we aim to understand the effect of NPC spectator group size on VR exergames to gather further insights into how to better incorporate this factor to improve players' gameplay performance and experience.

### 2.3. Effect of feedback

According to the social cognitive theory, people who are receiving encouraging messages tend to increase their effort to accomplish their objectives, resulting in positive outcomes and increased self-efficacy and self-confidence (Bandura, 1982, 1997, 2001). These messages often serve to provide individuals with support for their efforts. When this feedback is full of encouragement, it can inform their recipients that their performance has achieved a standard and made progress (Ilgen et al., 1979; Latham and Locke, 1991).

As for exercising, individuals who receive positive feedback typically experience heightened self-efficacy and improved performance (Fitzsimmons et al., 1991; Escarti and Guzman, 1999) as well as increased competence and intrinsic motivation (Gernigon and Delloye, 2003; Bindarwish and Tenenbaum, 2006). Although negative feedback might undermine an individual's belief in their ability and reduce their expectations of success (Fishbach et al., 2010), studies argue that negative feedback may boost goal-congruent behaviors as it signals to the individual that there is a lack of progress or his/her goal has not yet been achieved (Locke and Latham, 1991; Kluger and DeNisi, 1996; Fishbach et al., 2006, 2010).

In digital games, players who receive feedback based on their performance have more favorable gameplay experiences such as greater enjoyment, more motivation to play in the future, higher self-efficacy, and better game ratings (Peng et al., 2012). Kappen et al. (2014) have found that both positive and negative spectator feedback could increase game engagement for players more than a spectator that is silent. Kim and Timmerman (2016) suggested that players who received encouraging messages had greater enjoyment. To date, the literature has effectively documented the positive effects of feedback from a human spectator(s) in exergames. Nevertheless, whether feedback from NPC spectators matters is not clear (Kappen et al., 2014; Kim and Timmerman, 2016).

In our research, we delivered both visual and audio feedback as the literature suggests it could contribute to greater enjoyment and energy expenditure (Kim et al., 2014). In addition, we included encouraging feedback for both the player and the opponent in the game using both visual and audio cues because their combination represents more natural real-life scenarios.

## 3. Testbed: VR exergame

**Figure 1:**
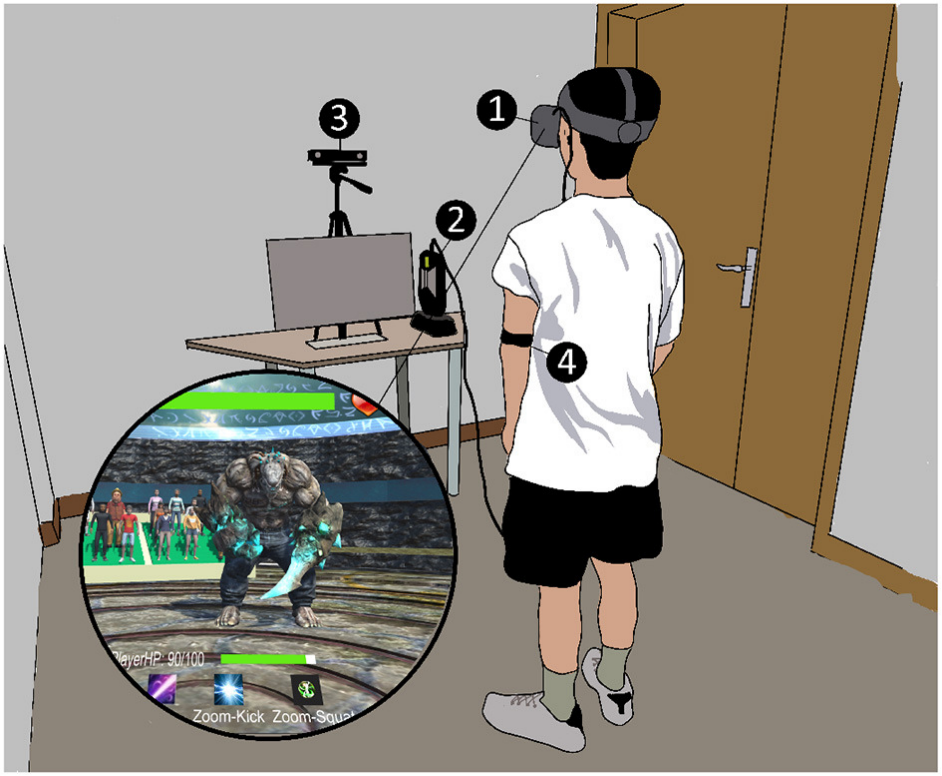
A picture of the exergame, apparatus, and setup of the experiment: (1) An Oculus Rift S; (2) An HP Z backpack workstation; (3) the Microsoft Kinect 2; and (4) Polar OH1. The computer monitor is only used for opening VR software purposes and did not have a direct role in the experiment.

For this research, we employed a modified version of the VR exergame GestureFit (Xu et al., 2021b) as our testbed environment. As Figure 1 shows, the game is a first-person view environment and requires the player to perform body-based gestures to make attacks (i.e., *Push, Kick, Zoom+Kick* gestures) against an opponent in the form of a non-human monster and defend (i.e., *Zoom+Squat* gestures) themselves from being attacked by it. Table 1 shows a summary of the gesture-based skill moves (or simply skills), their functions, and their requirements. In addition, we included features such as false-attack that could be performed by the monster to “trick” the player into doing a defense move to make the game more engaging (Malone, 1982; Costikyan, 2013; Kumari et al., 2019; Xu et al., 2021b).

**Table 1:** Description of each gesture move (or skill) by the player^a^ and the monster^b^, with instructions for how to perform the gesture, features, and requirements.

**Name**	**Instruction of the gesture**	**Function of the move**	**Cooldown**
*Kick* ^a^	Single leg kicking	Attack: inflict 10 HP damage to the opponent in the kicking direction	3 s
*Push* ^a,b^	Single hand pushing	Attack: inflict 10 HP damage to the opponent on the punching direction	3 s
*Zoom+Kick* ^a^	leaning arms forward and stretching them out (Zoom) + Single leg kicking	Ranged attack: inflict 30 HP damage to the opponent in the attack range (1 m)	5 s
*Zoom+Squat* ^a^	leaning arms forward and stretching them out (Zoom) and Performing a squat	Defense: releases a sphere to protect the user for 2 s and heals 20 HP if it could successfully protect the player from the monster's attack	3 s
*Squat* ^b^	N/A not for the player	Ranged attack: deal 30 HP damage	5 s

^a^Indicates skills belong to the player. ^b^Indicates skills belong to the monster.

Both the monster and the player have three lives, where the player has 100 health points (HP) for each life and the monster has 500 HP. When the player's or monster's HP drops to 0, they would respawn a new life until the number of lives is 0. The goal is to defeat the monster three times (three lives) and the player needs to stay alive. All these parameters are kept consistent with prior work (Xu et al., 2021b).

In line with previous studies that have used the Kinect 2 to track body motions, we limited players' lateral movement so that participants' gestures can always be captured accurately (Ioannou et al., 2019; Xu et al., 2020c, 2021b). In addition, visual and audio feedback was provided to give a fuller range of sensory experiences to players.

**Figure 2:**
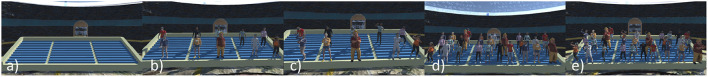
Conditions tested in our experiment: **(a)** *ES*: empty stadium; **(b)** *SSNF*: small NPC spectator group size without feedback to the player; **(c)** *SSWF*: small NPC spectator group size with feedback to the player; **(d)** *LSNF*: large NPC spectator group size without feedback to the player; **(e)** *LSWF*: large NPC spectator group size with feedback to the player. This figure only displays one spectator stand used in our game scene. There are three more spectator stands placed around the playfield or stadium.

Several modifications were made to adapt the original game to suit our research purposes. Specifically, we added a stadium to the game and four supporter stands where NPC spectators would be located. Figure 2 shows an example of each condition.

## 4. Study 1

### 4.1. Experiment design

Study 1 aims to explore whether NPC spectators could impact players' performance and experience in a gesture-based VR exergame. This Study followed a within-subjects design with two conditions (1) Empty Stadium (ES) and (2) Small Size with Feedback (SSWF). The order of these two conditions was counterbalanced.

### 4.2. Evaluated conditions

- *ES*: there were no spectators related feedback in the game (benchmark condition). See in the Figure 2a.
- *SSWF*: This condition deployed 40 NPC spectators in the game scene. The NPCs would react to both player's and opponent's behaviors. There were two types of feedback from these spectators that would be triggered by the following: (1) *Encouraging*: the player's supporters (30 NPCs) would cheer up when the player performed five successful consecutive actions based on combinations of attack and defend moves. (2) *Discouraging*: the opponents' supporters (10 NPCs) would cheer up when the opponent (i.e., the monster) performed three successful consecutive actions that could be a combination of attacks, defend moves, and false-attacks (that is, fake moves intended to trick the opponent into doing a defensive move). When any of these conditions were met, the respective spectators would cheer for the either player or monster using three types of motions (i.e., standing up, jumping, clapping of hands) with slightly adjusted time duration. Also, applause sounds mixed with cheering and hand clapping were provided. See in the Figure 2c.

The animation used for the cheer-up feedback is the same for the player and the monster. As we have arranged different locations for the supporters, the player can easily understand who their supporters are. The sound effect used is also the same; however, we have controlled the volume to differentiate the supporters so that it is aligned proportionally to their group sizes, with a higher volume for the player's supporters and a lower volume for the monster's supporters.

### 4.3. Instruments

For assessing the outcomes, we collected the following data:

- *Performance*: (1) completion time to finish the game, measured in seconds; (2) success rate of each gesture move; (3) the number of combo moves (simply combos from now) by the player; and (4) the number of combos by the monster. In our research, a combo is defined as a combination of successful performance moves. For the player, it is five consecutive successful actions based on a combination of attack and defend moves, while, for the monster, it is three consecutive successful actions that can be a combination of attack, defend, and false-attack moves.
  Players' performances of combos, defined as five successful actions in a row (attack+defend), could trigger encouraging behavior from the player's NPC supporters in conditions with Spectator Feedback, while the opponent's combos, three successful actions in a row (attack+defend+false-attack [i.e., to trick the player into making a defense move]), could trigger encouraging behavior from the monster's supporters. The sum of the player's combo and the sum of the opponent's combo could help us understand whether having encouraging behaviors could boost players' quality play and whether having discouraging behaviors could motivate players to prevent the monster from performing well in the game.
- *Experience*: We used a reduced 25-item modified version of the Game Experience Questionnaire (GEQ; Johnson et al., 2018) to measure participants' game experience (flow, immersion, competence, positive affect, negativity). The experience was rated based on a 5-Likert scale (where 0 indicates “not at all” and 4 indicates “extremely agreed”). This modified version was selected because it has higher reliability in comparison to the original version (IJsselsteijn et al., 2013).
- *Exertion*: (1) the average heart rate percentage (AvgHR%)—the average heart rate during the session divided by a participant's estimated maximum heart rate (211–0.64 × age; Nes et al., 2013) and (2) calories burned. Both heart rate and calories burned were monitored by Polar OH1, which has been proven to be a reliable HR measurement device (Schubert et al., 2018; Hettiarachchi et al., 2019).
- *Ranking and Comments*: We asked participants to rank the conditions based on their own preferences and provide comments on the games and feedback for improvement if they have any.

### 4.4. Hypotheses

A previous study suggests that having NPC spectators in a VR cycling game could improve players' performance and increase their heart rate (Haller et al., 2019). Therefore, we hypothesized that: **H1**: (a) There would be an improvement in players' (a) performance and (b) exertion (e.g., heart rate) when NPC spectators and their feedback were provided in the game.

In addition, because prior work in exergames with human spectator(s) suggests that both positive and negative spectator feedback could increase players' game engagement (Kappen et al., 2014), we predicted that: **H2**: Having NPC spectators and their feedback could result in a better game experience.

### 4.5. Apparatus and setup

Figure 1 shows the experiment setup and devices used in the experiment. An Oculus Rift S was used as our VR HMD. It was connected to an HP Z workstation with an i7 CPU, 16 GB RAM, and an Nvidia Quadro P5200 GPU. Players' gestures were detected via a Microsoft Kinect 2, which was also connected to the HP Z workstation. The heart rate (HR) and calories burned were monitored by a Polar OH1 optical HR sensor worn on the user's upper arm.

The experiment was conducted in an indoor laboratory room that was well-illuminated and could not be seen from the outside. The temperature of the room was controlled by an air conditioner that regulated the temperature to 24°C throughout the experiment.

### 4.6. Participants

Ten unpaid and physically healthy participants (six males and four females; mean age = 20.3, SD = 0.80, between 19 and 21; BMI = 21.72, SD = 2.83) were recruited from a local university campus through adverts such as message postings in a social network application and emails to a database of possible participants who had registered their interest in user studies or had participated in other similar user studies within the university. Six of them had experience with VR HMDs, and four had interacted with the Rift S before. However, none of them were regular VR users. Two played exergames before, but none of them was a regular exergame player. They all declared to be physically fit and had normal or corrected-to-normal vision.

We excluded participants who may suffer health risks from doing exercises through Physical Activity Readiness Questionnaire (PARQ) (Thomas et al., 1993) and who have resting heart rate (RestHR) level that was *too low* (i.e., females aged between 16 and 19: <62 beats/min and between 20 and 39: <60 beats/min; or males aged between 16 and 19: <56 beats/min and between 20 and 39: <55 beats/min), or *too high* (i.e., females aged between 16 and 19: >94 beats/min or between 20 and 39: >89 beats/min; or males aged between 16 and 19: RestHR >87 beats/min and between 20 and 39: >84 beats/min; Ostchega et al., 2011).

### 4.7. Procedure and task

The experiment lasted about 20 min for each participant, including 10 min playing the exergame. At the beginning of the experiment, participants needed to report their demographic information (e.g., age, gender, and experience with VR HMDs) and their health status via the PARQ. After a brief description of the experimental conditions (duration, gameplay, and location of their supporters and those of the monster in the environment) and procedure, participants signed the consent to participate in the experiment. We then asked participants to enter their personal information (e.g., age, gender, height) into the Polar Beat app and collected their RestHR.

Prior to each condition, an experimenter would help participants wear the Rift S. We only entered the experimental gameplay phase when participants' HR reached the equivalent RestHR level and they felt rested. After each condition, participants needed to fill in GEQ. Between conditions, participants were allowed to rest as much as they wanted and only proceeded to the next condition when they felt rested and their HR reached the equivalent resting level. They needed to rank all conditions and provide comments on conditions after they completed all conditions.

Although the local area had a minimum number of COVID-19 cases for 12 months, before the experiment we sanitized the device before and after each participant's turn and followed extra safety measures to ensure the safety of the participants and researchers (e.g., wearing a mask and staying at a safe distance and providing good ventilation).

## 5. Study 1 results

We used Shapiro-Wilks tests and Q-Q plots to check for violations of the normality of the data for all analyses. All tests reported were with two-tailed *p*-values.

For data that were normally distributed, we employed the one-way repeated measures with Conditions as the only independent variable (ES and SSWF); otherwise, we processed the data via Aligned Rank Transform (ART; Wobbrock et al., 2011) before we employed the one-way repeated measures ANOVA. Bonferroni corrections were used for all pairwise comparisons. We reported effect size as $\eta_{p}^{2}$ whenever there was a significant effect.

### 5.1. Game completion time

**Figure 3:**
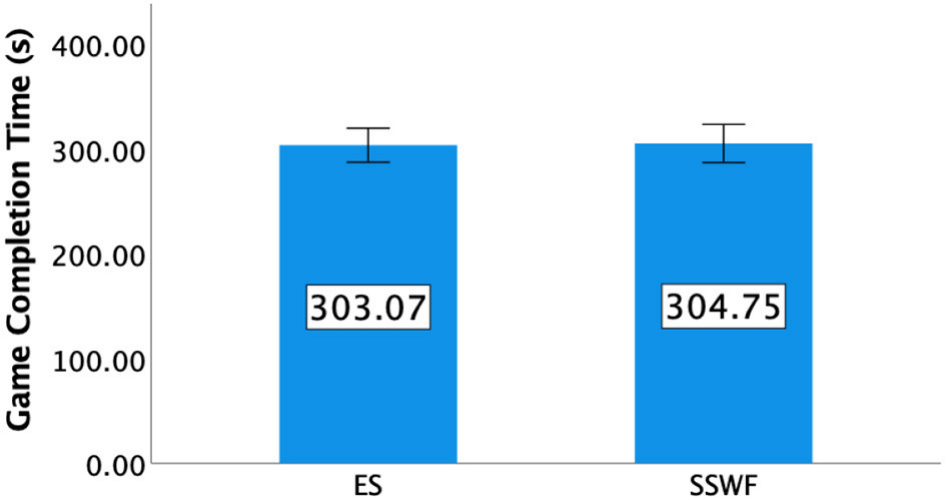
Mean game completion time in seconds for each condition in Study 1. Error bars indicate 95% confidence intervals.

One-way ANOVA yielded no significant difference between Conditions regarding game completion time [*F*_(1,9)_ = 0.072, *p* = 0.795] between ES (M = 303.1, SD = 22.68) and SSWF (M = 304.7, SD = 25.39). Details of game completion time can be found in Figure 3.

### 5.2. Success rates of gestures

#### 5.2.1. Push

**Figure 4:**
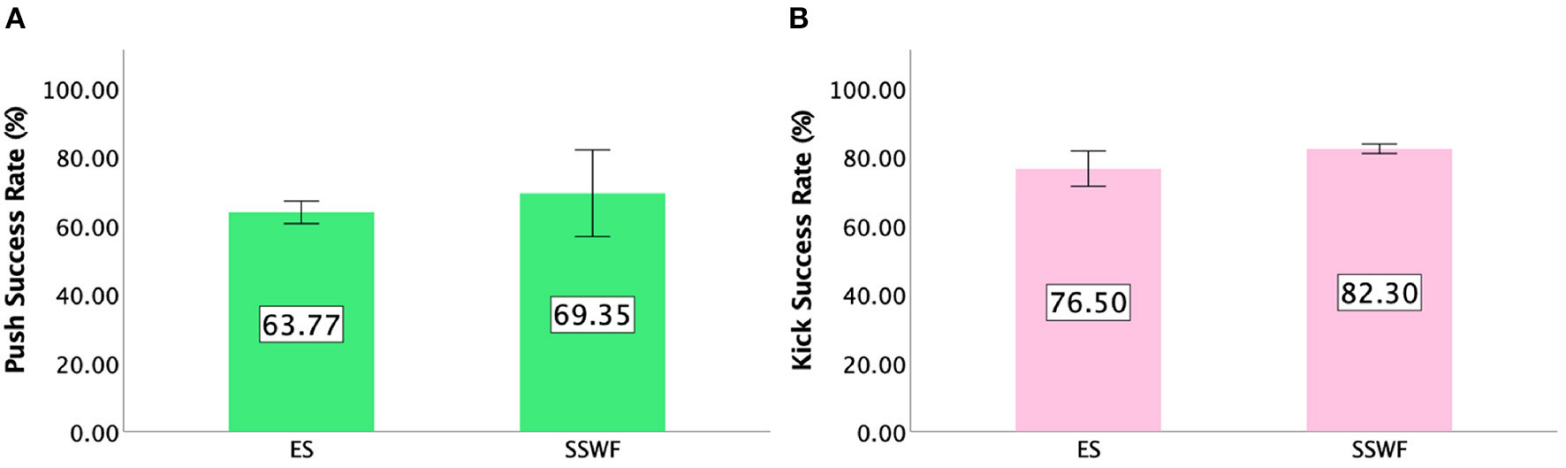
**(A)** Mean success rate of *Push*. **(B)** Mean success rate of *Kick* in Study 1. Error bars indicate 95% confidence intervals.

Details of the *Push* success rates can be found in Figure 4A. We could not find any significant difference between Conditions [*F*_(1,9)_ = 7.939, *p* = 0.358] on *Push* success rates.

#### 5.2.2. Kick

ANOVA tests showed a significant difference between Conditions [$F_{\left(1,9\right)}=7.221,p<0.05,\eta_{p}^{2}=0.445$]. *Post-hoc* tests indicated that players had higher *Kick* success rates when NPC spectators and their feedback were provided (*p* < 0.05). Details of the *Kick* success rates can be found in Figure 4B.

#### 5.2.3. Zoom+Kick

**Figure 5:**
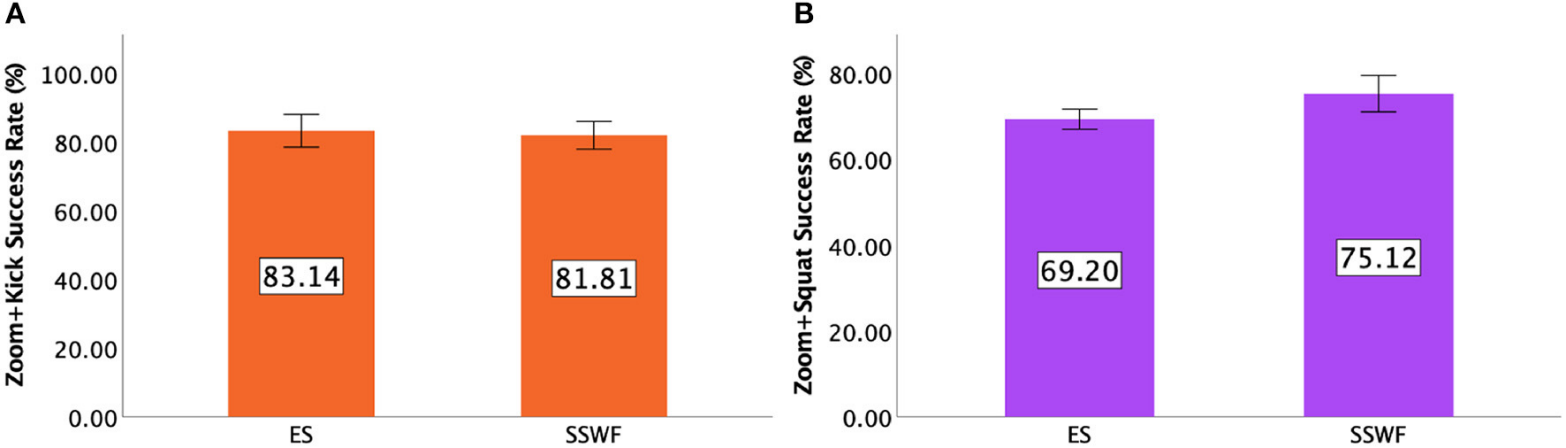
**(A)** Mean success rate of *Zoom+Kick*. **(B)** Mean success rate of *Zoom+Squat* in Study 1. Error bars indicate 95% confidence intervals.

An ANOVA indicated that there was no significant difference between Conditions [*F*_(1,9)_ = 0.681, *p* = 0.431]. Details of the *Zoom+Kick* success rate can be found in Figure 5A.

#### 5.2.4. Zoom+Squat

Figure 5B shows the *Zoom+Squat* success rates for each condition. An ANOVA yielded a significant difference between Conditions [$F_{\left(1,9\right)}=7.034,p<0.05,\eta_{p}^{2}=0.439$]. *Post-hoc* pairwise comparisons suggested that players had higher *Zoom+Squat* success rates when having spectators and their feedback in the scene (*p* < 0.05).

### 5.3. Combo performance

#### 5.3.1. Players' combos

**Figure 6:**
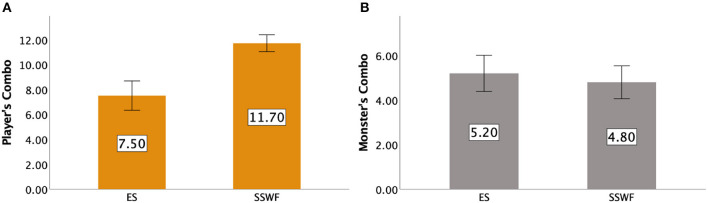
The average total number of Combos made by **(A)** the player and **(B)** the monster for each condition in Study 1. Error bars indicate 95% confidence intervals.

We observed a significant difference between Conditions [$F_{\left(1,9\right)}=47.250,p<0.001,\eta_{p}^{2}=0.840$] where *post-hoc* results suggested that participants performed more Combos when NPC spectators and their feedback were provided (*p* < 0.001). Figure 6A shows the details of players' Combo moves.

#### 5.3.2. The Monster's combos

Figure 6B shows the details of the monster's Combos. An ANOVA yielded no significant difference between Conditions [*F*_(1,9)_ = 0.643, *p* = 0.443].

### 5.4. Players' experience: GEQ

**Figure 7:**
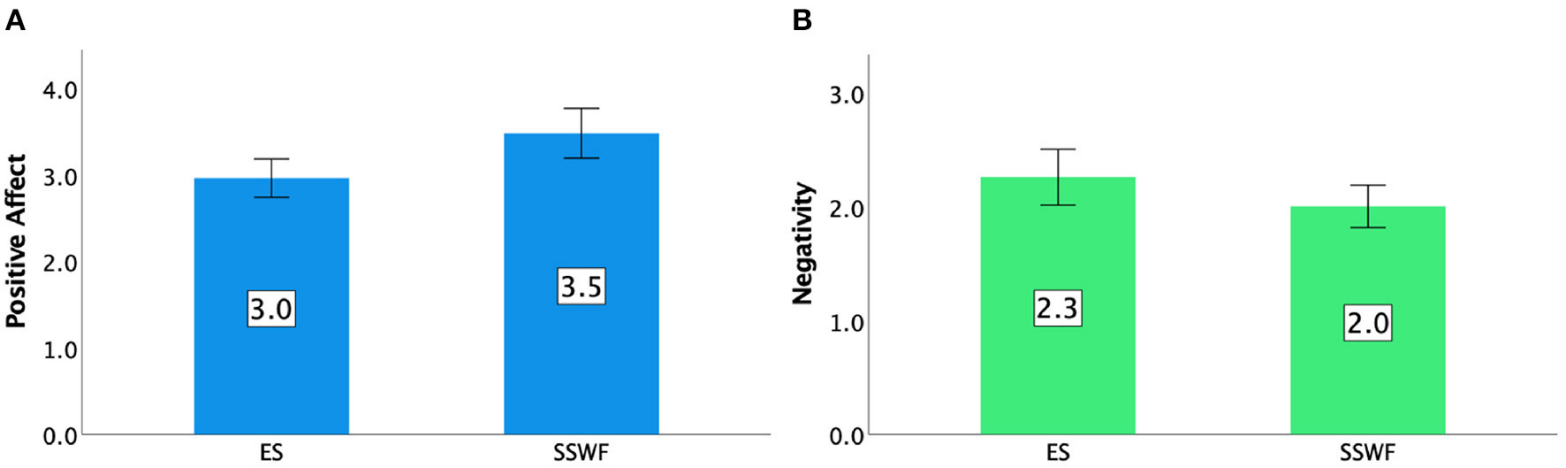
GEQ ratings in Study 1: **(A)** Positive Affect and **(B)** Negativity. Error bars indicate 95% confidence intervals.

**Figure 8:**
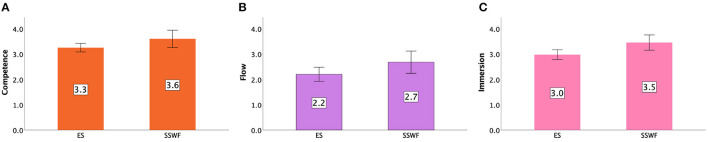
GEQ ratings in Study 1: **(A)** Competence, **(B)** Flow, and **(C)** Immersion. Error bars indicate 95% confidence intervals.

Each player's game experience in conditions was measured by GEQ in terms of Positive Affect, Negativity, Competence, Flow, and Immersion. Details can be found in Figures 7, 8.

#### 5.4.1. Positive affect

ANOVA tests showed that there was a significant difference between Conditions [$F_{\left(1,9\right)}=13.112,p<0.01,\eta_{p}^{2}=0.593$]. *Post-hoc* tests revealed that having NPC spectators and their feedback (SSWF) led to a higher level of Positive Affect than ES (*p* < 0.01).

#### 5.4.2. Negativity

We could not find any significant effect of the presence of a small number of NPC spectators and their feedback on Negativity ratings [*F*_(1,9)_ = 3.407, *p* = 0.098]. Negativity ratings can be seen in Figure 7B.

#### 5.4.3. Competence

An ANOVA yielded a significant difference between Conditions [$F_{\left(1,9\right)}=4.366,p<0.05,\eta_{p}^{2}=0.327$]. *Post-hoc* results indicated participants had higher Competence scores in SSWF than ES (*p* < 0.05; see Figure 8A).

#### 5.4.4. Flow

We found a significant difference between Conditions [$F_{\left(1,9\right)}=15.070,p<0.005,\eta_{p}^{2}=0.626$]. *Post-hoc* results indicated that participants had a better flow rating in SSWF than ES (*p* < 0.005). More details can be found in Figure 8B.

#### 5.4.5. Immersion

There was a statistically significant difference between Conditions regarding Immersion [$F_{\left(1,9\right)}=8.352,p<0.05,\eta_{p}^{2}=0.481$]. *Post-hoc* results showed that participants had a better immersion experience when playing the SSWF condition (*p* < 0.05) (see Figure 8C).

### 5.5. Players' exertion

**Figure 9:**
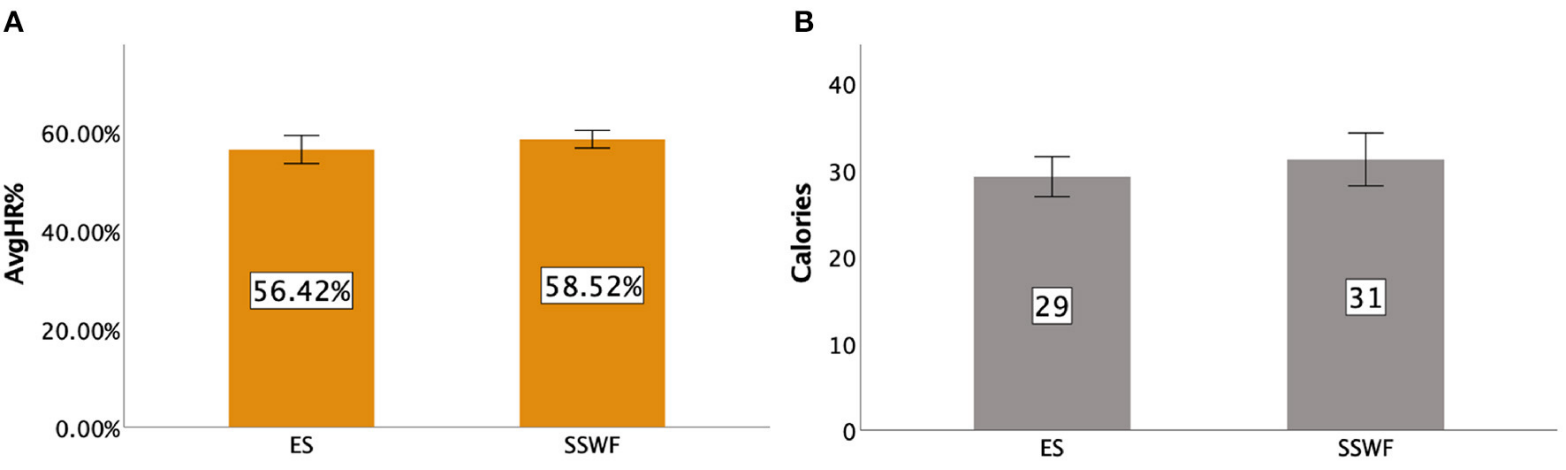
Exertion: **(A)** AvgHR%. **(B)** Calories burned for each condition in Study 1. Error bars indicate 95% confidence interval.

Figure 9 presents the details of AvgHR% and Calories Burned for each Condition. An ANOVA showed a significant difference between Conditions [$F_{\left(1,9\right)}=7.715,p<0.01,\eta_{p}^{2}=0.474$] on AvgHR%, and *post-hoc* tests revealed that participants had a higher AvgHR% when NPC spectators and their feedback were provided (*p* < 0.01).

For Calories burned, we found a significant difference between Conditions [$F_{\left(1,9\right)}=6.923,p<0.05,\eta_{p}^{2}=0.435$]. *Post-hoc* pairwise comparisons revealed that participants burned more calories with the SSWF condition (*p* < 0.05).

### 5.6. Ranking and qualitative feedback

All participants (*N* = 10) voted SSWF as their preferred game version. We labeled their participant ID as P1–P10 in the description below to show their comments. Overall, participants perceived the design of the NPC spectators with their feedback in the VR exergame as “*interesting/good*” and liked this design (P1, P3, P5-6, P8, P10). They felt that the game became “*competitive*” when the NPC spectators and feedback were presented in the game together (P1, P3, P5-6, P10). Some of them also said that the “*the size/the number of NPC characters is small and can be increased to make the game environment more interesting* (P9-10).”

### 5.7. Study 1 discussion on hypotheses

We found support in our results for **H1a** and **H1b**, where having NPC spectators improved participants' performance (i.e., better success rates in some gestures and performed more successful Combo moves) and increased their exertion level (i.e., higher AvgHR%). We confirmed that having NPC spectators and their feedback not only works in a VR cycling game (Haller et al., 2019) but also in gesture-based VR exergames.

We also found support for **H2**; that is, having NPC spectators and their feedback in VR exergames could enhance players' game experience as they gave higher ratings in several game experience measurements (i.e., positive affect, competent, flow, and immersion).

In conclusion, our Study 1 confirmed that it is beneficial to have NPC spectators and their feedback in gesture-based VR exergames, as it could improve participants' game performance, enhance their game experience, and increase their exertion levels. Interestingly, participants commented that it might be better if more NPCs can be present in the game scenes (i.e., a larger NPC spectator group size). As mentioned in Sections 2.2, 2.3, both NPC Spectator Group Size and Feedback are potential factors that can affect users' performance and gameplay experiences. Thus, we conducted a second study to explore further the role of NPC spectator group size and their feedback in VR exergames.

## 6. Study 2

### 6.1. Experiment design

In this study, we investigated two characteristics of NPC spectators (1) *NPC Spectator Group Size* (Small and Large) and (2) *Spectator Feedback* (With and Without). The experiment followed a two-way within-subjects design with these two characteristics as independent factors. The order of these four conditions was counterbalanced in the experiment.

### 6.2. Evaluated conditions

- *Small Size With No Feedback* (SSNF): This condition included 40 NPCs, with 30 supporting the player, which is the same as the SSWF condition in Study 1. However, the only difference between the SSNF and SSWF is the spectator in SSNF would not respond to either the player's or opponent's behaviors (see Figure 2b).
- *Small Size With Feedback* (SSWF): This condition is the same as the SSWF condition in Study 1 (see Figure 2c).
- *Large Size With No Feedback* (LSNF): This condition was analogous to SSNF. The only difference was that in this condition, there were 120 NPCs in the scene and 90 of them were supporting the player (see Figure 2d).
- *Large Size With Feedback* (LSWF): This condition was analogous to SSWF with two differences: (1) a larger NPC size (i.e., 120 NPCs), and (2) 50% louder sound effect of the spectator to reflect the increased size of the spectator (see Figure 2e).

Factors such as (1) the number of NPC spectators in small and large conditions (ranging from 30 to 200), (2) the percentage of the spectator supporting the player vs. the monster (e.g., 0, 25, 50, 75%), (3) the threshold of Combos (from 2 to 6), and (4) the spectator feedback, were all designed, developed, and validated through Rapid Iterative Testing and Evaluation with target participants (*N* = 5) in several rounds (Medlock, 2018). These five participants also helped identify any potential issues and recommend improvements (e.g., the volume of the feedback should be different because the size of the supporters was different). In addition, we confirmed that the player could easily see where the audience stands were located and could identify which audience size condition they were playing.

### 6.3. Apparatus, setup, and instruments

Study 2 used the same apparatus and setup as in Study 1. As for instruments, we employed all outcome measurements used in Study 1. In addition, we conducted a brief structured interview with open-ended questions asking them about their experience and performance toward the use of NPC-based spectators and their feedback in the game. The questions were: “*Overall, what did you think about the design of the spectators and their feedback on performance?*,” “*What did you like about the design of the spectators and their feedback on performance?*,” “*What did you not like about the design of the spectators and their feedback on performance?*” (Drachen et al., 2018). There was no limit to the length of participants' responses, which could be oral or written, if participants were not comfortable sharing their opinion verbally.

### 6.4. Hypotheses

The literature on the home choke effect suggests that players' performance might drop with the increase in NPC spectator group size, although not in all conditions (Böheim et al., 2019). The literature on VR public speaking tasks shows that a smaller number of spectators lead to significantly higher stress responses (in particular in heart rate) than a larger number of spectators (Mostajeran et al., 2020). Therefore, we predicted that: **H3**: There would be (a) a decrement in players' performance (b) but an increment in players' experience and exertion when the NPC spectator group size increases.

In line with our Hypothesis 2 in Study 1, as prior work in exergames with human spectator(s) suggests that both positive and negative spectator feedback could increase players' game engagement when compared to a silent spectator audience (Kappen et al., 2014), we hypothesized that: **H4**: Providing feedback could result in better game performance, game experience, and exertion.

### 6.5. Participants

Sixteen unpaid physically-abled participants (nine males and seven females; mean age = 20.06, SD = 0.77, range 19–21; BMI = 20.7, SD = 3.42) were recruited from the same local university campus as in Study 1 and using the same approach. Among them, eight had prior experience with VR HMDs, and five had interacted with the Rift S before. But none of them were frequent VR users. Seven participants played exergames before, but they were no regular exergame players. They all had normal or corrected-to-normal vision and self-declared to be physically fit. We employed the same exclusion process as described in Study 1. None of these participants had participated in Study 1.

### 6.6. Procedure and task

The procedure and task were consistent with Study 1; the only difference was that participants played four conditions in this study instead of 2. The experiment lasted about 45 min for each participant, with ~20 min of playing the exergame.

## 7. Results

We used Shapiro-Wilks tests and Q-Q plots to check for violations of the normality of the data for all analyses. All tests reported were with two-tailed *p*-values.

We used two-way repeated measures ANOVAs if the data were normally distributed; if the data were not normally distributed, we then would process the data through ART (Wobbrock et al., 2011) before using two-way repeated measures ANOVAs. Bonferroni corrections were used for all pairwise comparisons. We reported effect size using $\eta_{p}^{2}$ whenever there was a significant effect.

For open-ended questions from the structured interviews, answers were recorded and transcribed in text and later analyzed by two researchers following a thematic analysis (Braun and Clarke, 2006). The themes were concluded by the two researchers independently and agreed upon in a post-coding meeting with a third researcher.

### 7.1. Game completion time

**Figure 10:**
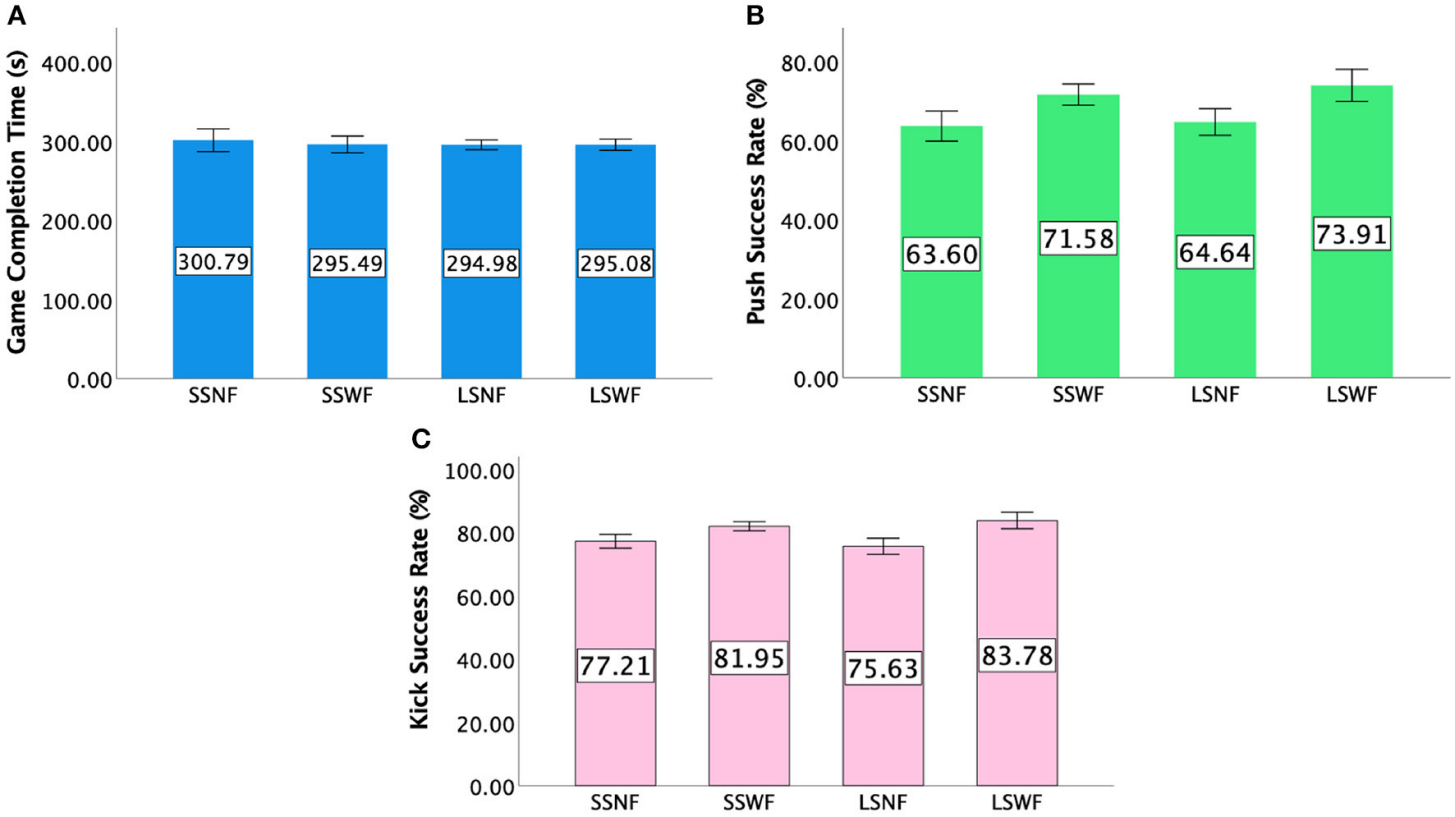
**(A)** Mean game completion time in seconds, **(B)** mean success rate of Push, and **(C)** mean success rate of *Kick* in Study 2. Error bars indicate 95% confidence intervals.

We could not find any significant effect of NPC Spectator Group Size [*F*_(1,15)_ = 0.421, *p* = 0.526] and Spectator Feedback [*F*_(1,15)_ = 0.402, *p* = 0.535] on the game completion time; no interaction was found either [*F*_(1,15)_ = 0.298, *p* = 0.593]. Details of game completion time can be found in Figure 10A.

### 7.2. Success rates of gestures

#### 7.2.1. Push

A repeated measures ANOVA yielded a significant main effect of Spectator Feedback [$F_{\left(1,15\right)}=33.886,p<0.001,\eta_{p}^{2}=0.693$] on *Push* success rates, but not NPC Spectator Group Size [*F*_(1,15)_ = 1.630, *p* = 0.221] nor interaction effect between NPC Spectator Group Size × Spectator Feedback [*F*_(1,15)_ = 0.115, *p* = 0.739]. *Post-hoc* pairwise comparisons showed that players had higher *Push* success rates when Spectator Feedback was provided than when it was absent (*p* < 0.001). Figure 10B shows the *Push* success rates for each condition.

#### 7.2.2. Kick

Two-way repeated measures ANOVA showed a significant main effect of Spectator Feedback [$F_{\left(1,15\right)}=64.739,p<0.001,\eta_{p}^{2}=0.812$], but no significant main effect of NPC Spectator Group Size [*F*_(1,15)_ = 0.12, *p* = 0.913]. In addition, we found a significant interaction effect between NPC Spectator Group Size × Spectator Feedback [$F_{\left(1,15\right)}=4.703,p<0.05,\eta_{p}^{2}=0.239$]. *Post-hoc* pairwise comparisons of the interaction effect showed participants had higher *Kick* success rates in SSWF than SSNF (*p* < 0.001) and in LSWF than LSNF (*p* < 0.001). Details of the *Kick* success rates results can be found in Figure 10C.

#### 7.2.3. Zoom+Kick

**Figure 11:**
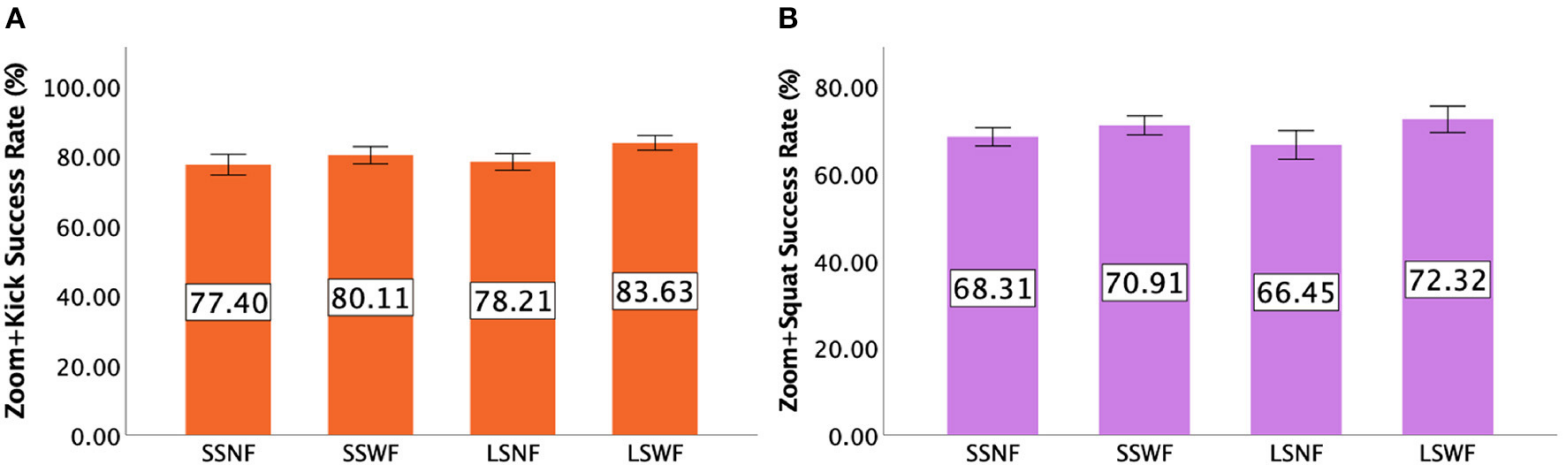
**(A)** Mean success rate of *Zoom+Kick* and **(B)** mean success rate of *Zoom+Squat* in Study 2. Error bars indicate 95% confidence intervals.

Two-way repeated measures ANOVA indicated that there was a significant main effect of NPC Spectator Group Size [$F_{\left(1,15\right)}=8.990,p<0.005,\eta_{p}^{2}=0.375$] and Spectator Feedback [$F_{\left(1,15\right)}=12.043,p<0.005,\eta_{p}^{2}=0.445$] but no significant interaction effect between NPC Spectator Group Size × Spectator Feedback [*F*_(1,15)_ = 1.120, *p* = 0.307]. *Post-hoc* pairwise comparisons indicated that players had higher *Zoom+Kick* success rates when NPC Spectator Group Size was large than it was small (*p* < 0.005) and Spectator Feedback was provided than without it (*p* < 0.005). Figure 11A shows the success rates of *Zoom+Kick* for each condition.

#### 7.2.4. Zoom+Squat

Figure 11B shows the *Zoom+Squat* success rates for each condition. Two-way repeated measures ANOVA yielded a significant main effect of Spectator Feedback [$F_{\left(1,15\right)}=17.927,p<0.001,\eta_{p}^{2}=0.544$], but no significant main effect of NPC Spectator Group Size [*F*_(1,15)_ = 0.026, *p* = 0.875] and no significant interaction effect between NPC Spectator Group Size × Spectator Feedback [*F*_(1,15)_ = 1.930, *p* = 0.185]. *Post-hoc* pairwise comparisons suggested that players had higher *Zoom+Squat* success rates when Spectator Feedback was provided than without it (*p* < 0.001).

### 7.3. Combo performance

#### 7.3.1. Players' combos

**Figure 12:**
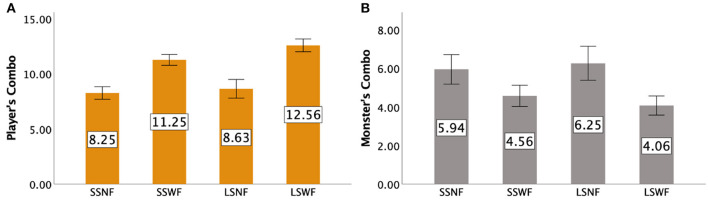
The average total number of Combos made by **(A)** the players and **(B)** the monster for each condition in Study 2. Error bars indicate 95% confidence intervals.

Repeated measures ANOVA tests indicated a significant main effect of NPC Spectator Group Size [$F_{\left(1,15\right)}=8.826,p<0.01,\eta_{p}^{2}=0.370$] and Spectator Feedback [$F_{\left(1,15\right)}=189.554,p<0.001,\eta_{p}^{2}=0.927$], but no significant interaction effect was found (*F*_(1,15)_ = 3.705, *p* = 0.073]. *Post-hoc* results suggested that players performed more Combos when NPC Spectator Group Size was large than when it was small (*p* < 0.01) and when feedback was provided than when feedback was absent (*p* < 0.001). Figure 12A shows the details of players' Combo moves.

#### 7.3.2. The Monster's combos

Figure 12B shows the details of the monster's Combos. Repeated measures ANOVA tests yielded a significant main effect of Spectator Feedback [$F_{\left(1,15\right)}=24.979,p<0.001,\eta_{p}^{2}=0.625$], but not NPC Spectator Group Size [*F*_(1,15)_ = 0.123, *p* = 0.730] and the interaction effect of NPC Spectator Group Size × Spectator Feedback [*F*_(1,15)_ = 1.610, *p* = 0.224]. *Post-hoc* results suggested that players prevented more of the monster's Combo moves when feedback was provided than when it was absent (*p* < 0.001).

### 7.4. Players' experience: GEQ

**Figure 13:**
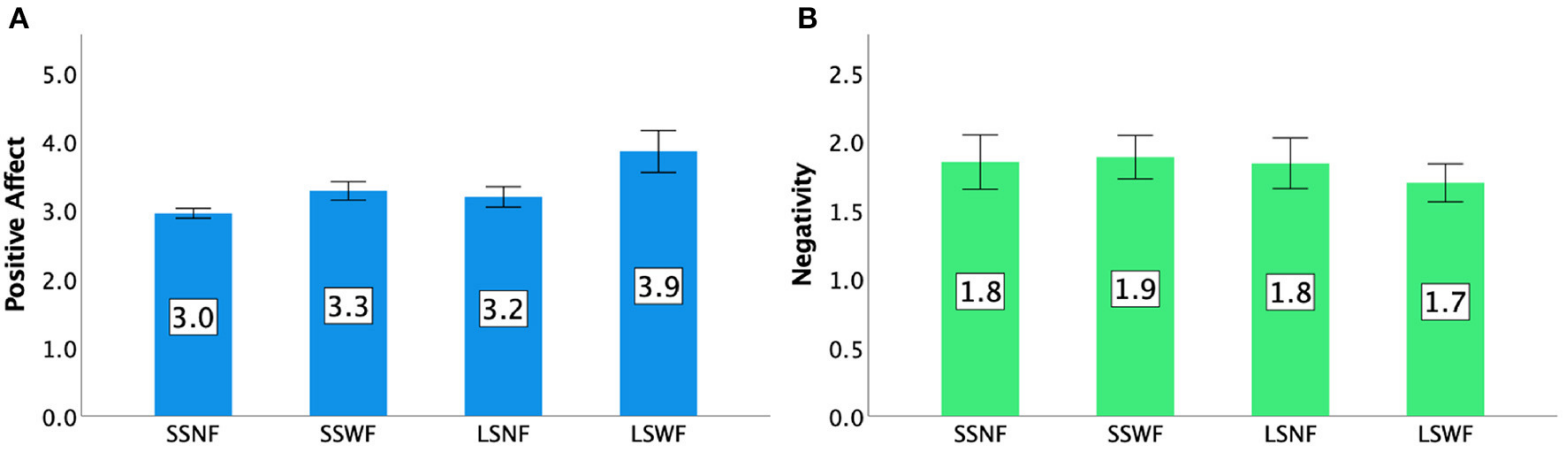
GEQ ratings in Study 2: **(A)** Positive Affect and **(B)** Negativity. Error bars indicate 95% confidence intervals.

**Figure 14:**
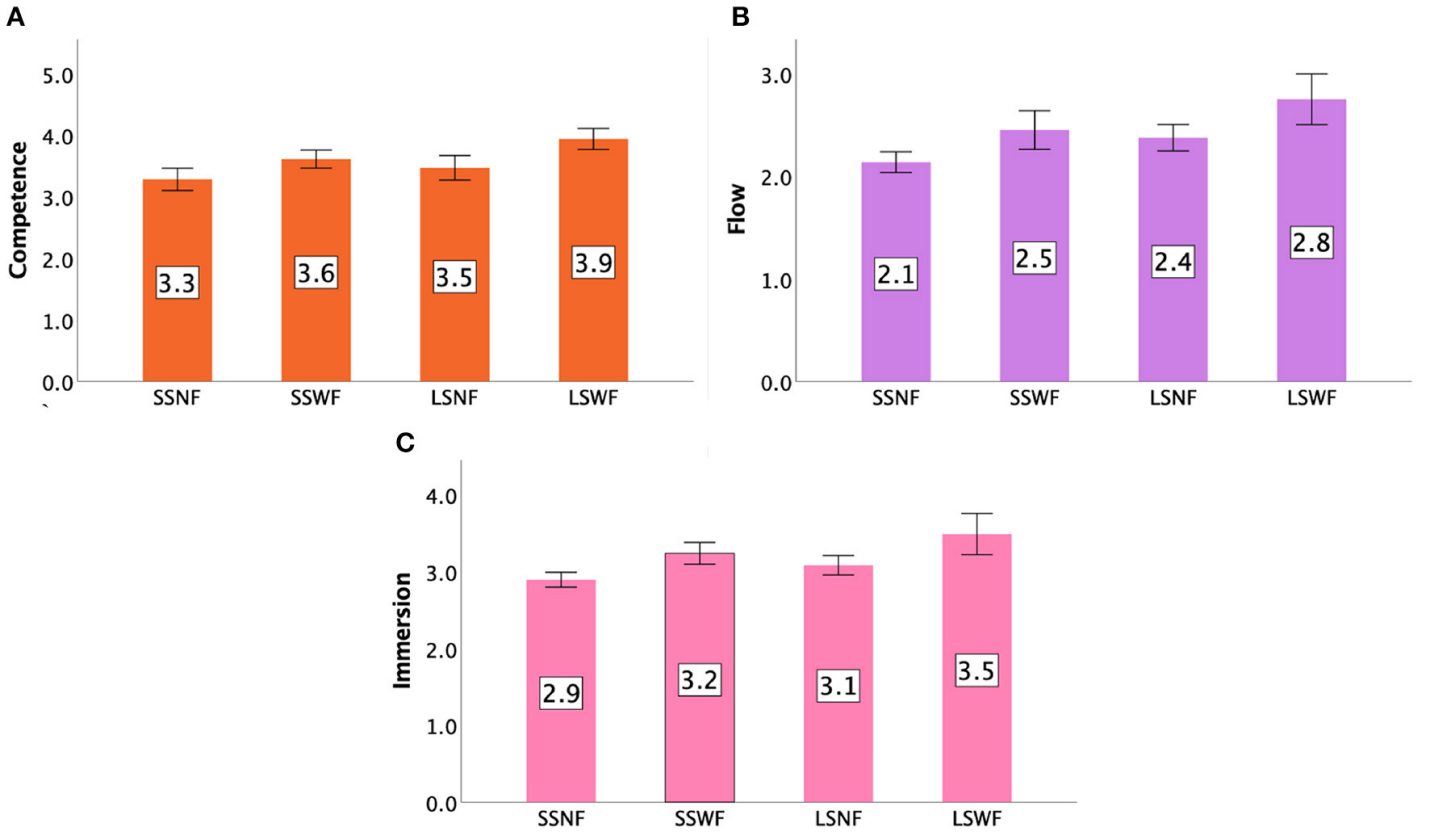
GEQ ratings in Study 2: **(A)** Competence, **(B)** Flow, and **(C)** Immersion. Error bars indicate 95% confidence intervals.

Values for each condition for Positive Affect, Competence, Flow, and Immersion can be found in Figures 13, 14.

#### 7.4.1. Positive affect

A two-way repeated measures ANOVA showed that both NPC Spectator Group Size [$F_{\left(1,15\right)}=29.795,p<0.001,\eta_{p}^{2}=0.665$] and Spectator Feedback [$F_{\left(1,15\right)}=31.983,p<0.001,\eta_{p}^{2}=0.681$] had significant main effects on Positive Affect. We also observed an interaction effect between NPC Spectator Group Size × Spectator Feedback [$F_{\left(1,15\right)}=12.328,p<0.005,\eta_{p}^{2}=0.451$]. *Post-hoc* pairwise comparisons based on the interaction effects suggested that (1) LSWF led to a greater Positive Affect than LSNF (*p* < 0.001) and SSWF (*p* < 0.001), (2) SSWF led to a greater Positive Affect than LSNF (*p* < 0.001) and SSNF (*p* < 0.001), and (3) LSNF led to a greater Positive Affect than SSNF (*p* < 0.001).

#### 7.4.2. Negativity

Repeated measures ANOVA tests indicated a significant main effect of Spectator Feedback [$F_{\left(1,15\right)}=6.901,p<0.05,\eta_{p}^{2}=0.315$], but not NPC Spectator Group Size [*F*_(1,15)_ = 1.262, *p* = 0.279] and the interaction effect between NPC Spectator Group Size and Spectator Feedback [*F*_(1,15)_ = 3.947, *p* = 0.066]. *Post-hoc* results confirmed that players had lower Negativity scores when Spectator Feedback was provided than when the feedback was absent (*p* < 0.05).

#### 7.4.3. Competence

Repeated measures ANOVA tests yielded significant main effects of NPC Spectator Group Size [$F_{\left(1,15\right)}=13.624,p<0.005,\eta_{p}^{2}=0.476$] and Spectator Feedback [$F_{\left(1,15\right)}=22.487,p<0.001,\eta_{p}^{2}=0.600$], but no interaction effect was found between NPC Spectator Group Size and Spectator Feedback [*F*_(1,15)_ = 2.238, *p* = 0.155]. *Post-hoc* results indicated that players had higher Competence scores (1) when the NPC Spectator Group Size was large than when it was small (*p* < 0.005) and (2) when Spectator Feedback was provided than when feedback was absent (*p* < 0.001).

#### 7.4.4. Flow

Repeated measures ANOVA tests showed that there were significant main effects of NPC Spectator Group Size [$F_{\left(1,15\right)}=24.265,p<0.001,\eta_{p}^{2}=0.618$], Spectator Feedback [$F_{\left(1,15\right)}=15.938,p<0.001,\eta_{p}^{2}=0.515$], but no interaction effect was found [*F*_(1,15)_ = 0.338, *p* = 0.570]. *Post-hoc* pairwise comparisons based on the interaction effects showed that players had higher Flow scores (1) in the large NPC Spectator Group Size condition than in the small one (*p* < 0.001) and (2) in the condition with feedback than the one without it (*p* < 0.001).

#### 7.4.5. Immersion

Repeated measures ANOVA tests yielded significant main effects of NPC Spectator Group Size [$F_{\left(1,15\right)}=9.304,p<0.01,\eta_{p}^{2}=0.383$] and Spectator Feedback [$F_{\left(1,15\right)}=17.419,p<0.001,\eta_{p}^{2}=0.537$], but no interaction effect was found [*F*_(1,15)_ = 0.484, *p* = 0.497]. *Post-hoc* results revealed that players had higher Immersion scores (1) when NPC Spectator Group Size was large than when it was small (*p* < 0.01) and (2) when Spectator Feedback was provided than when it was absent (*p* < 0.001).

### 7.5. Players' exertion

**Figure 15:**
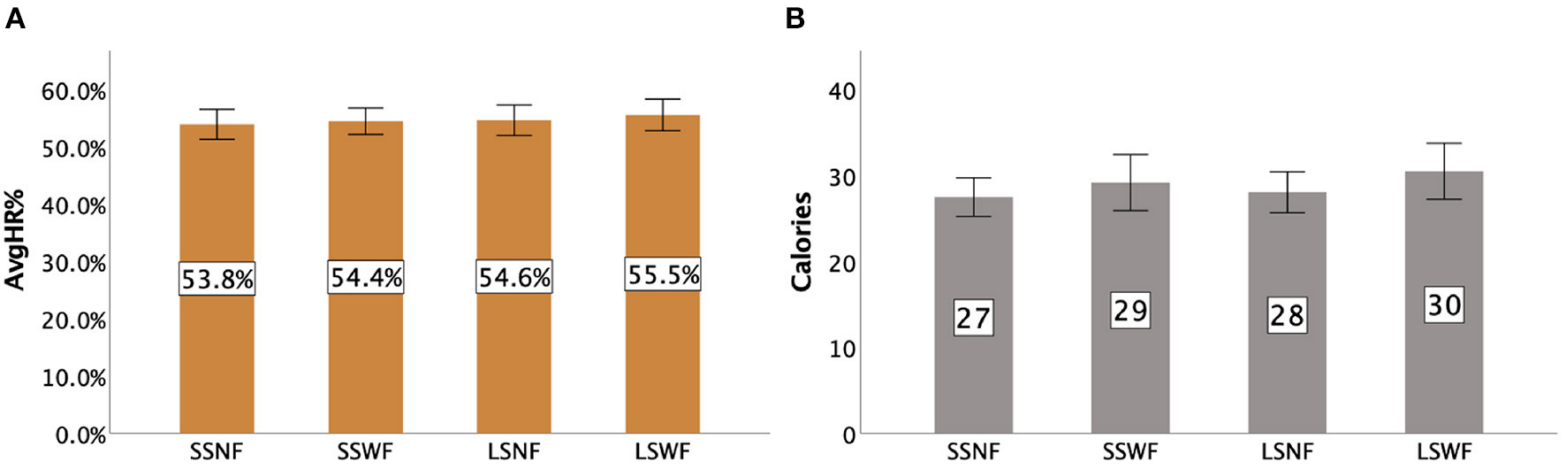
Exertion: **(A)** AvgHR%. **(B)** Calories Burned for each condition in Study 2. Error bars indicate 95% confidence intervals.

Figure 15 presents the details of AvgHR% and Calories Burned for each condition. Two-way repeated measures ANOVA tests yielded that there were significant main effects of NPC Spectator Group Size [$F_{\left(1,15\right)}=7.734,p<0.05,\eta_{p}^{2}=0.340$] and Spectator Feedback [$F_{\left(1,15\right)}=7.633,p<0.05,\eta_{p}^{2}=0.249$] on AvgHR%, but no significant interaction effect between NPC Spectator Group Size × Spectator Feedback [*F*_(1,15)_ = 0.435, *p* = 0.524] was found. *Post-hoc* pairwise comparisons revealed that participants had a higher AvgHR% when NPC Spectator Group Size was large than when it was small (*p* < 0.05) and Spectator Feedback was provided than when it was not given (*p* < 0.05).

For Calories Burned, we found a significant main effect of Spectator Feedback [$F_{\left(1,15\right)}=8.011,p<0.05,\eta_{p}^{2}=0.348$], but not of NPC Spectator Group Size [*F*_(1,15)_ = 1.431, *p* = 0.250] and NPC Spectator Group Size × Spectator Feedback [*F*_(1,19)_ = 0.344, *p* = 0.567]. *Post-hoc* pairwise comparisons revealed that participants burned more calories when Spectator Feedback was provided than when the feedback was absent (*p* < 0.05).

### 7.6. User ranking and feedback

The LSWF version was rated the best version among the four versions by all the participants (*N* = 16). There was a mix of votes between SSWF and LSNF as their second-best choice: 10 players voted for SSWF as their second option, and six voted for LSNF. All participants voted SSNF as the fourth option.

From the coded transcripts, two main themes emerged (*general gaming experience* and *suggestions for spectator design*) from two researchers, who first reviewed the transcripts independently. They were agreed upon by a third researcher after a second discussion. The 16 participants are labeled as P1–P16 in the description below.

Overall, players perceived the design of the spectators and their feedback in the VR exergame as “*interesting/useful*” (P1, P3-4, P6, P10-11, P14-16). They believed that having spectators and their feedback makes the game “*more enjoyable and competitive*” (P1, P3-4, P6, P9-10, P14-16).

Regarding the elements that they liked about the design of the spectators and their feedback in the VR exergame, in general, they liked the presence of these NPC spectators and their feedback “*it is nice to have fans to support me and their feedback are encouraging*” (P1-4, P7-11, P13, P15-16). They also thought that having spectators for both players and the monster was a good idea “*(it is good to have) spectators supporting both the player and the opponent*” (P1, P6, P11, P16). They also liked that “*the spectators have varied reactions to their performance*” (P5, P15). Some participants mentioned that they liked this aspect because it makes them feel competitive “*I felt more competitive when the NPC Spectator Group Size was large*” (P4, P9, P11, P13-16).

Regarding the elements that they disliked about the design of the spectators and their feedback in the VR exergame, most participants disliked the fact that the spectators were “*computer-controlled*” (P3, P5-6, P8, P11, P12-13, P15-16). In addition, they would like to see more variety of reactions/animations from the spectators, “*(the spectators) have a limited cheer up animations*” (P7, P9, P14, P16).

## 8. Discussion

### 8.1. Effect of spectator NPC group size on VR exergames

Our results indicate that having a large number (size) of NPC spectators surrounding the players could boost their combos and increase *Zoom+Kick* gesture success rate than having a smaller NPC spectator group size. In addition, we did not find any decrement in players' performance when the NPC spectator group size increased. Therefore, **H3a** was not supported. We found support in our results for **H3b**, where players had a more extraordinary positive game experience (Competence, Flow, Immersion) and a higher overall AvgHR% when the NPC spectator group size was large. In summary, a larger NPC spectator group size (1) does not affect players' performance negatively, not supporting (Böheim et al., 2019); instead, it enhances players' combo performance and increases *Zoom+Kick* gesture success rate in the gesture-based VR exergame, (2) affects players' experience positively, leading to a greater positive game experience and higher heart rate.

### 8.2. Effect of spectator feedback on VR exergames

The purpose of having spectator feedback on players' performance is to give affirmative messages to their actions, showing them that their performance has achieved a certain standard and that they are making progress (Ilgen et al., 1979; Latham and Locke, 1991), which should result in positive outcomes (e.g., self-efficacy and self-confidence; Bandura, 2001). Our results show that this element has helped achieve its intended goal because participants felt more skillful in conditions with feedback from the spectators than without it. In addition, we observed that this affirmation has increased players' performance (i.e., higher success rates of gestures, more combos performed, more monster's combos prevented), enhanced their overall experience (positive affect, competence, flow, and immersion), reduced the negative game experience, led to a higher AvgHR%, and burned more calories. Therefore, the **H4** was supported. In summary, having spectator feedback could positively affect players' performance, experience, and exertion. These findings are aligned with previous work on exercises (Fitzsimmons et al., 1991; Deci et al., 1999; Escarti and Guzman, 1999), digital games (Kappen et al., 2014), and non-VR exergames (Kim and Timmerman, 2016) when feedback is provided to players.

In short, our findings show that the presence of a virtual spectator audience and its feedback on the player's actions are very important to VR exergames. Specifically, they present a clear case for VR exergames to include a large number of NPC spectators with supportive feedback (for both the player and the monster) since they could improve the overall gameplay performance and experience. From a psychology perspective, our work is important because enjoyment and performance can be affected by users' perception of environmental factors. In the case of VR exergames, a virtual audience is one such factor, just like a real audience of spectators in a sports match. As such, the findings of our work can support designers in identifying suitable features for a virtual audience to help enhance users' perception of their abilities and hence their performance and enjoyment.

We next provide two design recommendations based on the findings and our observations from this investigation.

### 8.3. Design recommendations

#### 8.3.1. Enabling spectator feedback as a main priority

Based on the social cognitive theory, people who receive encouraging messages tend to increase their effort to accomplish their objectives, resulting in positive outcomes (Bandura, 2001). Our study has confirmed that having spectator feedback based on players' actions could boost their performance and enhance their game experience. Therefore, we suggest that exergame designers/researchers should include some type of spectator support and encouraging messages whenever players are making good progress, for instance, (1) when their performance has achieved a certain level (e.g., combos moves), (2) when their performance has resulted in good outcomes (e.g., taking the lead in rounds won), and (3) when their performance has prevented the opponent from performing well (e.g., blocking an opponent's action). This feature should be considered regardless of the group size of the spectator audience—as our results showed, even a relatively small number of spectators (*N* = 40) has led to a positive effect on players' performance and gameplay experience.

#### 8.3.2. Adding larger spectator audiences

**Figure 16:**
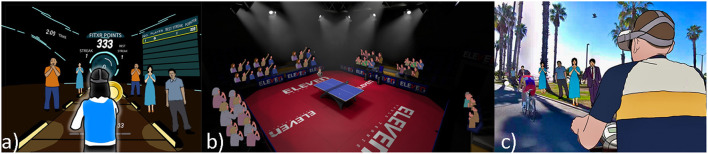
Examples of how NPC spectators can be added to games based on the degree of locomotion **(a)** *Low*, **(b)** *Medium*, and **(c)** *High* the player has in VR.

Our results appear to indicate that a larger number of spectator NPCs has the potential to enhance the gameplay experience. We list some examples of how the spectator NPCs can be added to the other exergames according to the degree of locomotion the player has in VR: (1) *Low*: in exergames that the player mostly remains at the same location [e.g., VirusBoxing (Xu et al., 2020b), Beat Saber^4^], spectators/supporters can be added behind the player, alongside the objects' flying area, or placed at a higher location where the game objects are initialized. (2) *Medium*: in exergames where players only need to move in a limited in-game distance (Eleven Table Tennis VR^5^), spectators can be added around the users but with a small distance. A good example is the Creed: Rise to Glory. (3) *High*: in exergames where players need to move their in-game position on a large scale (e.g., VR cycling games; Barathi et al., 2018), the spectator could be placed alongside the playing or track field (e.g., placing the spectators next to the street for VR cycling games). See examples in Figure 16.

### 8.4. Limitations and future work

One limitation is related to the spectators in that we only involved three standard types of cheering motions (i.e., standing up, jumping in place, clapping of their hands) and only one type of sound (mixture of cheering and hand clapping). Future work could (1) involve more types of cheering motions—hands up, dancing, thumb up, waving flags, and more sounds—singing songs and screaming, (2) adjust the length/frequency of the cheering to make them dynamic, and (3) add facial expressions to the spectators as a type of performance feedback. Our study only employed 10 character models and while our participants did not have any issues with the relatively homogeneous spectators, future work can involve more models with more diverse cultural and physical features to see if they have the same effect on players' performance and gameplay. Models could even be non-human (e.g., aliens or robots) or similar to famous characters in movies of whom players are fans. Our study involves encouraging feedback for both the player and the monster. The encouraging feedback for the monster might be perceived as negative (or neutral) by the players. Hence, our study could only confirm that the mixture of both feedback types could improve gameplay performance and experience. Understanding the effect of more specific types of feedback (such as encouraging/positive and discouraging/negative) is interesting and helpful to the design of effective and enjoyable exergames. In addition to the types of feedback, it would be interesting to examine the effect of players' familiarity with the audience, for example, by having virtual characters that look like the players' friends, relatives, and people with whom they are acquainted, similar to research in other domains such as training tools (Monteiro et al., 2020a, 2023). We have plans to conduct this research in the future.

Another limitation is that an experimenter's presence might have impacted players' performance and experience (Emmerich and Masuch, 2018). However, this cannot be avoided because there is a risk that participants could get injured while playing a VR exergame (Xu et al., 2020a). Having an experimenter onsite is a safety measure in experiments involving exergames. Although the presence of the experimenter could not be avoided, we have the experimenter presented in all conditions. As such, the potential effect of the experimenter on the players is balanced across the conditions, which should not affect our results.

Our study involved a relatively small sample size. While the sample size is within the range of similar studies involving VR games of different types (e.g., Gerling et al., 2020; Monteiro et al., 2020b; Wang et al., 2022a,b), it would be useful for future research to include more participants and with greater diversity (e.g., with/without physical impairments; Gerling et al., 2020; Creed et al., 2023). In addition, we provided participants with some level of rest as a washout period to let residual effects dissipate, as they were allowed to rest as much as they wanted and could only do the next condition when their heart rate was in the same rest heart rate level. On the other hand, it will be interesting to have experiments that allow more resting time in case the side effects are not fully vanished. For example, it is possible to design experiments lasting over two or more days to allow exploring the long-term use of exergames, similar to Xu et al. (2021a).

While gender differences were not a focus of this work, we conducted further analysis to see whether gender impacted players' performance using a three-way mixed ANOVA with Gender as a between-subjects factor. We found there was a significant impact of Gender on the Monster's Combos [*F*_(1,14)_ = 12.858, *p* < 0.01], where female participants (M = 4.68, SD = 1.36) prevented more Monster's Combos than male participants (M = 5.611, SD = 1.61). We could not observe any other significant differences. Further work can be conducted to investigate the impact of gender on participants' performance in VR exergames in more detail.

Finally, while this work is focused on exergames, the findings can also be used for other types of applications [e.g., for language training and learning (Pack et al., 2020; Barrett et al., 2023) and abstract concept exploration (Lu et al., 2018, 2023; Chen et al., 2021)] where the presence of a spectator audience can have an impact on users' performance. Still, future studies could explore using spectator NPCs other than exergames, where spectators can be added to give more realism to the applications.

## 9. Conclusion

In this work, we first confirmed that having NPC spectators in gesture-based VR exergames could improve game performance, experience, and exertion. Then, we further explored two characteristics of the spectators—NPC spectator group size (small/large) and the spectator's feedback (with/without). Our results led to two important observations for the type of exergame used in our research (1) a large number of spectators was more helpful since it could improve the overall game experience (higher competence, flow, immersion), increases AvgHR%, and would not negatively affect the performance (improved players' combo performance and increased gesture success rate for certain gesture); (2) spectator feedback was very useful in improving players' performance (higher gesture success rates, more combos performed successfully, more monster's combos prevented), enhancing game experience (positive affect, competence, flow, and immersion), reducing negative game experience, increasing exertion (AvgHR% and burned more calories). Most importantly, having spectator feedback responding to players' actions led to a higher impact on players than simply providing NPC spectators in the game without any feedback, even with a large number of such spectators. From these results, we derived two main design recommendations which could pave the way for improving gameplay performance and experience of VR exergames among young adults.

## Data availability statement

Datasets are available on request: The raw data supporting the conclusions of this article will be made available by the authors, without undue reservation.

## Ethics statement

The studies involving human participants were reviewed and approved by University Ethics Committee. The patients/participants provided their written informed consent to participate in this study.

## Author contributions

All authors listed have made a substantial, direct, and intellectual contribution to the work and approved it for publication.

## Footnotes

^1^ https://nba.2k.com/

^2^ https://www.ea.com/en-gb/games/fifa/fifa-23

^3^ https://store.steampowered.com/app/804490/Creed_Rise_to_Glory/

^4^ https://beatsaber.com/

^5^ https://elevenvr.com/en/
